# Novel Therapeutic Development for Nasopharyngeal Carcinoma

**DOI:** 10.3390/curroncol32090479

**Published:** 2025-08-26

**Authors:** Jongwoo Kim, Yunjoo Lee, Seoin Kim, Jong Chul Park

**Affiliations:** 1 MetroWest Medical Center, Framingham, MA 01702, USA; 2 Medicine-Hematology and Medical Oncology, Massachusetts General Hospital, Boston, MA 02114, USA; 3 Department of Medicine, Harvard Medical School, Boston, MA 02115, USA; 4 Ragon Institute, Cambridge, MA 02139, USA

**Keywords:** nasopharyngeal carcinoma, immunotherapy, therapeutic vaccine, adaptive cell therapy

## Abstract

Nasopharyngeal carcinoma (NPC) is a rare malignancy with a distinct epidemiological pattern and is most often associated with Epstein–Barr virus (EBV). EBV plays a critical role in NPC pathogenesis, with viral proteins driving oncogenesis by altering immune regulation, apoptosis, and tumor progression. The unique molecular landscape of NPC presents both challenges and opportunities for therapeutic development, particularly in the recurrent and metastatic (R/M) setting, where treatment resistance remains a major hurdle. While platinum-based chemotherapy has traditionally been the standard of care for R/M NPC, immune checkpoint inhibitors (ICIs) have emerged as a key component of treatment. However, both intrinsic and acquired resistance to PD-1/PD-L1 blockade underscore the need for alternative strategies, including modulation of alternative immune checkpoints and simultaneous engagement of non-redundant pathways to enhance responses and durability. Leveraging EBV-driven biology, emerging immunotherapeutic approaches, such as EBV-specific adoptive cellular therapies and therapeutic vaccines, aim to induce durable immunity to viral proteins. Additionally, targeted therapies including receptor tyrosine kinase inhibitors, epigenetic modulators, and antibody–drug conjugates are redefining precision medicine by selectively delivering cytotoxic agents to tumors. With growing insights into the biology of NPC and evolving therapeutics, the integration of immunotherapy, targeted agents, and biomarker-driven strategies is poised to transform NPC treatment, emphasizing biology-driven, multimodal approaches to optimize patient outcomes.

## Simple Summary

Key objective: How is the treatment landscape for recurrent/metastatic nasopharyngeal carcinoma (NPC) changing, and what new therapies are emerging to overcome the limitations of current systemic treatments and target EBV-driven tumor biology? Knowledge generated: Recent advancements include next-generation immune-modulating agents, adoptive cell therapies, and EBV-targeted therapeutic vaccines. Antibody–drug conjugates and molecularly targeted agents are under investigation to enhance precision and overcome treatment resistance. Relevance: Emerging novel therapies have the potential to improve clinical outcomes in advanced NPC by targeting unique biology and addressing therapeutic resistance, thereby offering new options for patients who have limited standard treatment options.

## 1. Introduction

Nasopharyngeal carcinoma (NPC) is a malignancy arising from the nasopharyngeal epithelium, characterized by distinct epidemiologic patterns based on geography, sex, and histologic subtype. Although globally rare (~1 case per 100,000 annually), its incidence is markedly elevated in endemic regions such as Southern China and Southeast Asia compared to Western populations [1]. NPC exhibits a strong male predominance (~2.5:1) and typically presents between ages 40 and 60 [2]. The World Health Organization classifies NPC into three histologic subtypes: keratinizing squamous cell carcinoma (WHO type I), more common in non-endemic areas and often associated with smoking or HPV infection; and non-keratinizing carcinomas, predominant in endemic regions, further divided into differentiated (WHO type II) and undifferentiated (WHO type III) forms. The latter subtypes, which are exclusively associated with Epstein–Barr virus (EBV), exhibit greater radiosensitivity and generally have a more favorable treatment response [3]. Genetic susceptibility also plays a significant role, with human leukocyte antigen (HLA) haplotypes and other polymorphisms linked to increased risk [4].

### 1.1. Cancer Biology

EBV infection is a key etiologic factor in NPC, particularly in endemic regions [5]. EBV establishes a latent infection that evades immune clearance, during which the selective expression of viral oncogenic proteins and noncoding RNAs promotes cellular transformation and tumorigenesis [6,7]. Among the latent viral proteins, latent membrane protein 1 (LMP1) and latent membrane protein 2A (LMP2A) act as mimics of host receptors and activate oncogenic signaling pathways, including nuclear factor kappa B (NF-κB), mitogen-activated protein kinase (MAPK), and phosphoinositide 3-kinase/protein kinase B (PI3K–Akt) [8]. This promotes chronic inflammation and epithelial–mesenchymal transition (EMT), immune evasion, and cell survival [9]. Epstein–Barr nuclear antigen 1 (EBNA1) maintains the viral episome and can induce oxidative stress and interfere with DNA repair pathways, potentially contributing to genomic instability [10].

In addition to viral proteins, EBV expresses noncoding RNAs, further driving NPC progression. EBV-encoded small RNAs (EBER1 and EBER2) exhibit dual roles: they activate pattern recognition receptors (TLR3, RIG-I) to induce pro-inflammatory cytokines but also inhibit apoptosis, with context-dependent effects on tumorigenesis [7,11]. Similarly, microRNAs derived from the BamHI A rightward transcript (BART) cluster inhibit pro-apoptotic factors such as p53 upregulated modulator of apoptosis (PUMA) and Bcl-2 interacting mediator of cell death (BIM), while also promoting angiogenesis via regulation of vascular endothelial growth factor (VEGF) [12,13].

Moreover, host genetic and epigenetic alterations shape disease susceptibility and progression. Genome-wide association studies (GWAS) have identified risk loci within the human leukocyte antigen (HLA) region and other immune-related genes, implicating inherited predisposition [14,15]. Epigenetic modifications, particularly promoter hypermethylation, contribute to tumor progression by silencing tumor suppressor genes such as Ras association domain family 1 isoform A (RASSF1A) [16,17]. EBV infection induces these alterations by inducing aberrant DNA methylation and histone modification patterns in host cells [18].

While EBV-positive NPC dominates in endemic areas, EBV-negative NPC, more prevalent in non-endemic regions, is often linked to environmental factors like smoking and HPV infection. EBV-negative tumors exhibit distinct genomic profiles, including higher rates of TP53 mutations and lower immune infiltration [3]. These differences underscore the need for subtype-specific strategies based on the EBV association in therapeutic development.

Beyond traditional views of NPC centered on somatic mutations and gene alterations as primary drivers, recent paradigms challenge this view, proposing cancer as a complex ecological and evolutionary system [19,20]. The NPC ecology theory posits that NPC is an ecological disease, a multidimensional spatiotemporal unity of ecology and evolution among pathological ecosystems. In this framework, NPC cells act as invasive species, with metastasis representing multidirectional ecological dispersal involving intravasation, circulation, and colonization, shifting from linear reductionism to holistic frameworks that consider evolutionary dynamics and ecosystem interactions.

### 1.2. Staging and Prognosis

The American Joint Committee on Cancer (AJCC) TNM staging system has recently introduced key revisions in the ninth edition to improve disease classification and prognosis. A key update is the reclassification of radiologically defined nonmetastatic disease into stages I to III, defined as follows: Stage I includes T1–2N0–1, Stage II comprises T3 or N2 disease, and Stage III encompasses T4 or N3 disease. Metastatic disease is now designated as Stage IV, with further subdivisions into IVA (M1a, ≤3 metastatic lesions) and IVB (M1b, >3 lesions). Extranodal extension into adjacent structures (e.g., muscle, skin, or neurovascular bundles) is now recognized as an adverse prognostic feature under the N3 category [21]. Advanced imaging modalities, including PET/CT and radiomics-guided MRI, are increasingly used to refine prognosis and guide personalized treatment planning [22,23].

NPC prognosis is highly dependent on the stage at diagnosis, with early-stage disease having a five-year survival rate exceeding 90%, while advanced stages are associated with significantly worse outcomes [24,25]. The anatomical location of NPC deep in the nasopharynx, surrounded by critical structures, complicates early detection, leading to late-stage diagnoses in many patients [26]. Plasma EBV DNA has emerged as a validated prognostic biomarker, with higher pre-treatment levels associated with greater tumor burden and worse outcomes [27].

### 1.3. Current Standard Therapy

NPC is predominantly managed with non-surgical, radiation-intensive strategies. For early-stage disease (Stages I–II), definitive radiation therapy remains the standard of care, with prophylactic nodal irradiation. For Stage II, the addition of concurrent chemoradiation with cisplatin enhances treatment efficacy. Locally advanced disease (Stages III and IVA) requires a more aggressive approach, with induction chemotherapy followed by concurrent chemoradiation [28].

For recurrent or metastatic (R/M) NPC, treatment options remain limited, and the prognosis is poor. In carefully selected cases, endoscopic nasopharyngectomy may be a salvage option for locally recurrent NPC limited to the nasopharynx and adjacent superficial structures after multidisciplinary assessment [29]. In patients considered for re-irradiation for locally advanced recurrent NPC, the hyperfractionated approach may decrease late toxicity and improve survival compared with standard fractionation [30]. For the majority of R/M patients, local salvage is not feasible, and management relies on palliative systemic therapy. Current National Comprehensive Cancer Network (NCCN) guidelines recommend the combination of anti-programmed cell death protein 1 (PD-1) immune checkpoint inhibitors (ICIs), such as toripalimab and penpulimab, with platinum-based chemotherapy as the preferred first-line therapy. If ICIs were not included in the initial treatment regimen, they are recommended upon disease progression following platinum-based chemotherapy. Subsequent options include second-line cytotoxic agents. However, targeted therapies such as cetuximab, an epidermal growth factor receptor (EGFR)-targeting monoclonal antibody, have demonstrated limited clinical benefits [31].

Advances in tumor immunobiology and drug development are reshaping the therapeutic landscape of R/M NPC. Emerging strategies, such as next-generation immune checkpoint inhibitors, adoptive cell therapies, bispecific antibodies, antibody–drug conjugates (ADCs), and molecularly targeted agents, seek to improve tumor selectivity, modulate the immunosuppressive microenvironment, and overcome therapeutic resistance. Collectively, these modalities represent a rapidly evolving frontier in the management of R/M NPC.

## 2. Novel Therapeutics in NPC

### 2.1. Immune Cell-Activating Agents

**Figure 1:**
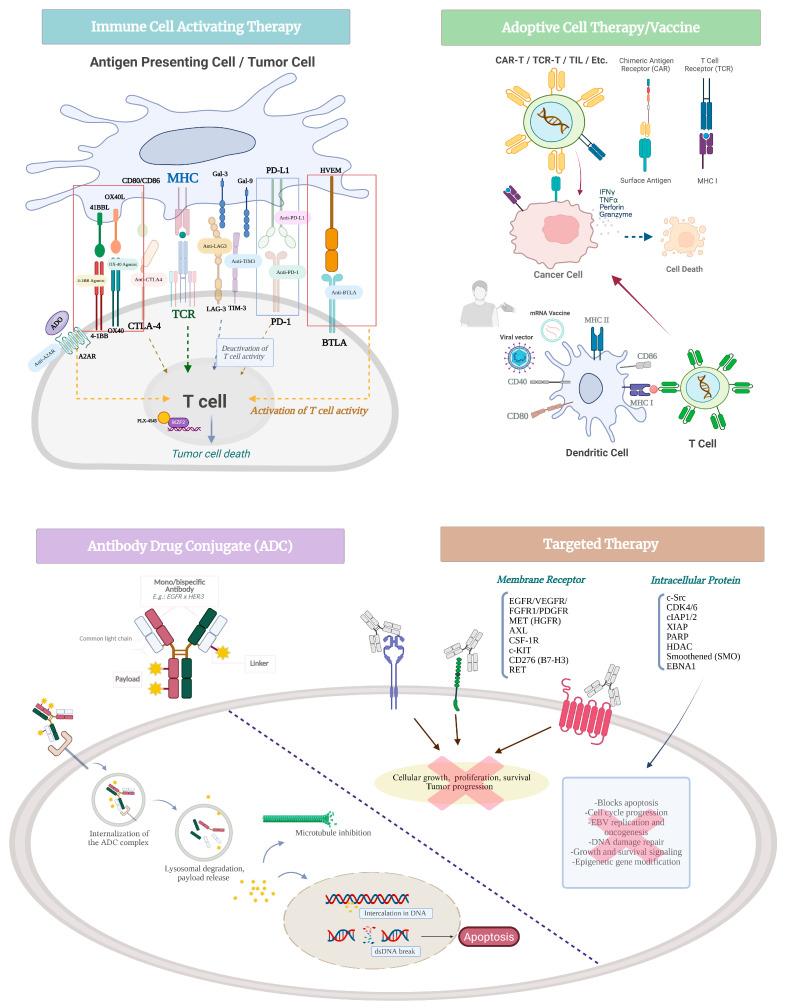
Mechanisms of action of novel agents in nasopharyngeal carcinoma. A2AR, Adenosine A2A receptor; ADO, Adenosine; BTLA, B- and T-lymphocyte attenuator; CAR-T, Chimeric antigen receptor T cells; CDK, Cyclin-dependent kinase; cIAP/XIAP, cellular/X-linked inhibitor of apoptosis protein; CSF-1R, Colony-stimulating factor 1 receptor; CTLA-4, Cytotoxic T-lymphocyte-associated protein 4; dsDNA, Double-stranded DNA; EBNA1, Epstein–Barr virus nuclear antigen 1; EGFR, Epidermal growth factor receptor; FGFR, Fibroblast growth factor receptor; Gal, Galectin; HDAC, Histone deacetylase; HGFR, Hepatocyte growth factor receptor; HVEM, Herpesvirus entry mediator; IFNr, Interferon-gamma; LAG-3, Lymphocyte-activation gene 3; MET, Mesenchymal–epithelial transition factor; MHC, Major histocompatibility complex; mRNA, Messenger RNA; OX40L, OX40 ligand; PARP, Poly(ADP-ribose) polymerase; PD-1, programmed cell death protein-1; PDGFR, Platelet-derived growth factor receptor; PD-L1, Programmed death-ligand 1; SMO, Smoothened receptor (Hedgehog pathway); TCR, T-cell receptor; TCR-T, T-cell receptor-engineered T cells; TIM-3, T-cell immunoglobulin and mucin-domain protein 3; TIL, Tumor-infiltrating lymphocytes; TNFα, Tumor necrosis factor-alpha; VEGFR, Vascular endothelial growth factor receptor; XIAP, X-linked inhibitor of apoptosis protein.

EBV-associated NPC is characterized by chronic antigenic stimulation from viral proteins (e.g., LMP1, EBNA1), which contributes to the development of a profoundly immunosuppressive tumor microenvironment (TME). This immunosuppression is driven by the accumulation of immunoregulatory cell populations, including regulatory T cells (Tregs), myeloid-derived suppressor cells (MDSCs), and tolerogenic macrophages, all of which inhibit effector T cell function and impair anti-tumor immunity [32]. These immunologic features, especially in the context of R/M disease, provide a compelling biological rationale for the use of ICIs and other immune-activating strategies in the treatment of NPC (Figure 1 and Table 1).

#### 2.1.1. Immune Checkpoint Inhibitors

##### PD-1/PD-L1 Inhibitors

Chronic exposure to EBV antigens leads to sustained PD-1 expression on tumor-infiltrating lymphocytes and upregulated programmed death ligand 1 (PD-L1) expression on tumor and stromal cells, particularly in nonkeratinizing subtypes of NPC. Monoclonal antibodies targeting PD-1, including toripalimab, tislelizumab, penpulimab, pembrolizumab, and nivolumab, are currently used to treat advanced NPC, either in combination with chemotherapy as first-line therapy or as monotherapy following platinum-based chemotherapy [31]. To improve treatment convenience, subcutaneous formulations such as envafolimab are being evaluated as alternatives to intravenous administration in locoregionally advanced NPC. Early-phase trials demonstrate promising antitumor activity and manageable safety when combined with chemoradiotherapy, though comparative efficacy against intravenous checkpoint inhibitors remains unstudied [33]. Combinatorial strategies involving anti-PD-1 or PD-L1 agents with immunomodulators or molecular targeted therapies are under investigation to enhance efficacy and overcome resistance, especially with anti-PD-1 refractory disease [34].

##### Beyond PD-1/PD-L1

Despite their clinical approval, PD-1 and PD-L1 inhibitors demonstrate only modest efficacy in NPC, with objective response rates of approximately 15–20% [35]. This limited clinical activity is partly attributed to the upregulation of alternative immune checkpoints that prevent sustained T-cell activation and induce adaptive immune resistance. Compensatory expression of co-inhibitory receptors, including cytotoxic T-lymphocyte-associated antigen 4 (CTLA-4), lymphocyte activation gene 3 (LAG-3), T-cell immunoglobulin and mucin domain 3 (TIM-3), and B- and T-lymphocyte attenuator (BTLA), has been implicated in T-cell exhaustion. These redundant inhibitory pathways undermine antitumor immunity and may limit the therapeutic efficacy of PD-1/PD-L1 blockade in NPC [36].

To address these resistance mechanisms, several early-phase combination strategies are investigating dual checkpoint blockade strategies. IBI310, an anti-CTLA-4 monoclonal antibody that competes with CD28 for B7 ligands and inhibits early T-cell activation, is being evaluated in combination with the PD-1 inhibitor sintilimab in patients with advanced solid tumors [37]. Similarly, LAG-3 blockade seeks to reverse T-cell dysfunction through co-inhibitory signaling disruption. LBL-007, a novel humanized anti-LAG-3 antibody, is currently being evaluated in combination with PD-1 inhibitors. Preliminary data suggest that this dual blockade approach may improve clinical outcomes in advanced NPC patients. LBL-007 and toripalimab combination showed an objective response rate (ORR) of 33.3% in ICI-native (*n* = 12) and an ORR of 11.8% (*n* = 17) in ICI-refractory NPC patients [38]. A randomized study is ongoing to test the benefit of relatlimab, an anti-LAG-3 monoclonal antibody, currently approved for the treatment of melanoma, added to anti-PD1 ICI as maintenance therapy after initial chemo-immunotherapy combination (REMAIN, NRG-HN011, NCT06029270). However, TIM-3 inhibitors such as TQB2618 have shown favorable safety and limited efficacy (N = 17, ORR 0%) [39]. Tifcemalimab (JS004), a monoclonal antibody targeting BTLA, is under clinical investigation in NPC and other solid tumors, but clinical data are not yet available [40].

##### Bispecific Immune Checkpoint Inhibitors

Bispecific immune checkpoint inhibitors are being developed to enhance antitumor immunity by simultaneously targeting multiple immune regulatory pathways. One promising approach involves dual blockade of PD-L1 and transforming growth factor beta (TGF-β). TGF-β is a cytokine frequently overexpressed in NPC, which contributes to immune evasion by promoting Treg differentiation and suppressing cytotoxic T cell function [41]. Bintrafusp alfa is a bifunctional fusion protein consisting of a monoclonal antibody targeting PD-L1 fused to the extracellular domain of TGF-β receptor II, designed to trap and neutralize TGF-β within the TME [42]. However, bintafusp alpha showed a modest single-agent activity with an ORR of 23.7% and concerning safety issues, including bleeding, anemia, and some cases of hyper-progression in platinum-refractory NPC patients [42].

Other PD-L1 and TGF-β dual-targeting agents, such as SHR-1701 and TQB2858, are currently under clinical evaluation, with early-phase studies indicating potential antitumor activity in NPC. Cadonilimab, a bispecific antibody targeting PD-1 and CTLA-4, has shown clinical activity with an ORR of 68% when combined with chemotherapy, which is similar to the standard anti-PD-1-platinum chemo combination, in patients with PD-1-refractory R/M NPC [43]. Additional bispecific antibodies, including SI-B003, QL1706 (also known as PSB205), and vudalimab (XmAb20717), are currently in early-phase clinical trials targeting solid tumors, including NPC. Preliminary findings from these studies indicate acceptable safety profiles and a single-agent efficacy with an ORR of 24.5% (N = 77) and 13% (N = 32), respectively [44,45,46].

#### 2.1.2. T Cell Co-Stimulatory Pathways

In contrast to immune checkpoint inhibitors, which suppress inhibitory signaling in effector T cells, co-stimulatory receptor agonists aim to deliver activating signals that amplify T cell responses. These agents target receptors of the tumor necrosis factor (TNF) receptor superfamily, such as OX40 (CD134) and 4-1BB (CD137), which are predominantly expressed following T cell activation [47,48]. Upon ligand binding or agonist engagement, these receptors initiate intracellular cascades mediated by TNF receptor associated factor (TRAF) adaptors. This leads to the activation of the NF-κB and MAPK pathways, which promote T cell proliferation, cytokine secretion (such as interleukin-2 (IL-2) and interferon gamma), and enhanced survival of effector and memory T cell subsets [49]. BAT6026, an agonistic anti-OX40 antibody, and ADG106, a 4-1BB agonist, have been tested in phase I trials including small NPC patient cohorts [50,51]. To date, no co-stimulatory agonist has demonstrated significant clinical activity in NPC.

#### 2.1.3. Other Immune Modulating Agents

Metabolic dysregulation within the TME of NPC promotes immune evasion by creating an immunosuppressive milieu dominated by lactate accumulation, adenosine signaling, and lipid metabolic rewiring, presenting actionable targets for immunomodulatory agents [52,53]. A key component of this process is the accumulation of tumor-derived adenosine, which activates the A2A receptor (A2AR) on T cells, leading to the suppression of their effector functions [54]. To counteract this pathway, ILB-2109, a selective A2AR antagonist, is being developed to restore T cell activity in adenosine-rich environments by blocking this immunosuppressive signaling [55]. In addition, chronic EBV antigen exposure fosters an immunosuppressive TME enriched with Tregs that exhibit upregulated IKZF2 (Helios), a transcription factor critical for maintaining their suppressive function. Helios enforces immunosuppression by epigenetically repressing IL-2 transcription and stabilizing Foxp3 [56]. PLX-4545, an oral IKZF2 degrader, is under clinical investigation to reprogram immunosuppressive Tregs into effector-like cells. Preclinical studies demonstrate synergy with PD-1 inhibitors, potentially enhancing antitumor immunity by alleviating Treg-mediated suppression [57].

### 2.2. Therapeutic Vaccine

Therapeutic vaccination represents a promising strategy for NPC given its strong etiologic link to EBV. Unlike rapidly mutating tumor antigens, EBV latency proteins remain genetically stable and consistently expressed, enabling robust epitope-specific vaccine design. Multiple vaccine modalities are currently under investigation in both clinical and preclinical settings (Figure 1 and Table 1).

Dendritic cell (DC) vaccines involve ex vivo loading of autologous DCs with EBV peptides (e.g., LMP2), followed by reinfusion to enhance antigen-specific immunity [58]. These vaccines activate both CD8^+^ and CD4^+^ T cells through major histocompatibility complex (MHC) class I and II pathways, demonstrating safety, albeit with modest immunogenicity [59]. Viral vector-based vaccines, including modified vaccinia Ankara (MVA-EL) encoding EBNA1 and LMP2, as well as recombinant adenoviral vectors targeting LMP2, have demonstrated target antigen-specific T cell responses, but clinical anti-tumor efficacy data are limited [60,61].

Peptide vaccines targeting LMP2 epitopes elicit detectable CD8^+^ responses, although their efficacy remains limited by HLA restriction and low immunogenicity. DNA-based vaccines encoding full-length EBV antigens, often fused to immunostimulatory elements such as CD40 ligand, have demonstrated enhanced antigen presentation and T cell activation in preclinical models; however, clinical validation remains pending [62,63]. Messenger RNA (mRNA)-based platforms have recently emerged as a novel approach to induce EBV-specific T-cell responses in NPC. Preclinical studies using lipid nanoparticle–encapsulated LMP2 mRNA (e.g., LPX-mLMP2) and early-phase clinical candidates such as WGc-043 have demonstrated robust CD8^+^ T-cell activation, reduced plasma EBV DNA, and signs of disease control, supporting their therapeutic potential [64].

The viral antigenic stability and restricted expression of EBV latency proteins in nonkeratinizing NPC make them ideal targets for vaccination [65]. However, viral immune evasion mechanisms, including EBNA1-mediated inhibition of MHC I presentation, limit effective antigen processing [66]. While no clinical trials have yet combined vaccines with PD-1/PD-L1 inhibitors, preclinical data suggest synergistic potential [67].

### 2.3. Adoptive Cell Therapy

Recent advances in T-cell engineering have broadened the scope of cancer immunotherapy beyond checkpoint inhibition and vaccination. Adoptive cell therapy (ACT), which involves the transfer of ex vivo expanded or genetically modified T cells, enables direct augmentation of antitumor immunity. Unlike vaccine-based approaches that depend on endogenous priming, ACT provides immediate delivery of antigen-specific cytotoxic T cells [68] (Figure 1 and Table 1).

EBV-specific cytotoxic T lymphocytes (EBV-CTLs) were among the earliest forms of ACT explored in NPC [69]. These autologous T cells are expanded ex vivo to recognize latent EBV antigens such as LMP1, LMP2, and EBNA1, and retain the ability to target tumor cells despite the restricted antigen profile of latency II [70]. Early-phase trials demonstrated favorable safety and preliminary clinical activity [71]. However, the Phase III randomized study, VANCE, of adding autologous EBV-CTL to standard chemotherapy failed to show a survival benefit compared to chemotherapy alone in patients with R/M EBV+ NPC [72]. Additionally, broader clinical application has been constrained by individualized manufacturing, variable antigen expression, and T-cell dysfunction in heavily pretreated patients.

To overcome these limitations, allogeneic EBV-CTLs derived from healthy donors have been developed as scalable, off-the-shelf alternatives that circumvent prior therapy–induced immunosuppression [73]. However, risks such as allo-reactivity and HLA mismatch require careful donor selection and monitoring. Tabelecleucel is an allogenic EBV-CTL, approved in Europe for the treatment of EBV-mediated post-transplant lymphoproliferative disease. Tabelecleucel has also been tested in EBV+ NPC population, but its efficacy was limited with no objective responses, and its development in this population has been terminated [74].

While the adoptive transfer of tumor-infiltrating lymphocytes (TILs), harvested from the tumor, has demonstrated feasibility when administered with IL-2 following chemoradiotherapy in patients with locally advanced NPC, the contribution of TILs to the overall clinical activity is difficult to determine [75].

Chimeric antigen receptor (CAR)-T cell therapy represents a next-generation ACT platform, enabling MHC-independent recognition of tumor-associated surface antigens [76]. Although early constructs have primarily targeted EBV antigens, development remains at an early stage due to manufacturing complexity and limited scalability. To broaden target coverage, novel surface antigens such as epithelial cell adhesion molecule (EpCAM) and CD70 are being investigated [77].

T-cell receptor (TCR) engineered T cells leverage native MHC-restricted antigen recognition to target intracellular EBV antigens in NPC. Unlike CAR-T cells, which primarily engage surface proteins in an MHC-independent manner, TCR-T cells can detect low-abundance intracellular peptides with high specificity and affinity [78]. Hybrid platforms combining TCR and CAR architectures are being investigated to integrate the advantages of both recognition pathways and potentially reduce immune escape [79]. Although several early-phase clinical trials of TCR-T therapy are underway in NPC, clinical efficacy data are currently lacking.

Beyond traditional ACT modalities, novel strategies are being tested, including polyclonal autologous T cell expansions, which aim to generate broad immune responses, γδ T cell-based therapy, which recognizes stress ligands independently of MHC, making them suitable for tumors with impaired antigen presentation. The clinical testing of such strategies is still in an early stage, and the safety and efficacy have not yet been verified.

### 2.4. Antibody–Drug Conjugates

Antibody–drug conjugates (ADCs) represent a novel class of therapeutics that integrate the specificity of monoclonal antibodies with the cytotoxic potency of chemotherapeutic agents. Their modular design enables the targeted delivery of potent cytotoxins to tumor cells expressing specific surface antigens, thereby minimizing systemic toxicity [80,81] (Figure 1 and Table 1).

EGFR is overexpressed in a substantial proportion of NPC tumors and contributes to proliferation, metastasis, and treatment resistance via the activation of the PI3K/AKT and MAPK signaling pathways [82]. Anti-EGFR antibodies, such as cetuximab and nimotuzumab, have been evaluated in combination with chemoradiotherapy or chemotherapy, with variable efficacy but notable toxicities [83]. EGFR-targeting ADCs, such as becotatug vedotin, MRG003, represent a next-generation approach by coupling EGFR specificity with the delivery of potent cytotoxins like monomethyl auristatin E (MMAE) via receptor-mediated endocytosis. Becotatug vedotin achieved an ORR of 30.2% in R/M NPC patients who had received prior platinum chemo and anti-PD-1/PD-L1 therapy, compared to that of 11.2% with standard chemotherapy [84]. However, careful evaluation of safety profiles is warranted given the potential for on-target, off-tumor toxicity in normal EGFR-expressing tissues. Grade 3 or higher treatment-related adverse events with becotatug were seen in 45.3% of patients in this study.

CD70 is a member of the TNF ligand family and contributes to immune evasion in NPC by engaging CD27 on T cells [85]. While CD27 signaling plays a role in T-cell co-stimulation under normal immune responses, in the TME, it has been implicated in sustaining Treg survival and promoting immune suppression. GEN1160, an ADC targeting CD70, delivers a DNA-damaging topoisomerase inhibitor directly to tumor cells, leveraging DNA repair deficiencies commonly seen in virally driven tumors. This dual mechanism of disrupting immunosuppressive signaling via the CD70–CD27 axis and inducing genotoxic stress positions CD70 as an attractive therapeutic target currently being assessed in a first-in-human Phase I trial for advanced solid tumors, including NPC [86].

CD276 (B7-H3) is another immune checkpoint molecule overexpressed in various solid tumors. It modulates immune cell infiltration and promotes macrophage-mediated T cell suppression. The ADC YL201 is designed to engage CD276, employing a tumor-microenvironment-responsive linker to release its cytotoxic payload. This design enables selective cytotoxicity within the immunosuppressive TME, potentially reversing immune exclusion and enhancing anti-tumor immunity [87]. YL201 has demonstrated promising efficacy in multiple solid tumor types, including NPC, with an ORR of 48.6% (NPC cohort *n* = 70) [87].

To overcome limitations associated with single-antigen targeting, bispecific ADCs have been developed as a next-generation strategy to improve therapeutic precision and address antigen heterogeneity. For instance, BL-B01D1 simultaneously targets EGFR and HER3 and facilitates improved internalization efficiency [88,89]. BL-B01D1 showed an early sign of promising activity in NPC patients with a 38% ORR (*n* = 42) [89]. Similarly, GEN1286 incorporates dual specificity for EGFR and MET, two receptors frequently co-expressed in epithelial tumors. In NPC and other EGFR-driven cancers, compensatory MET activation has been implicated in resistance to EGFR-targeted therapies by reactivating downstream PI3K/AKT and MAPK signaling and promoting EMT. By co-targeting EGFR and MET, GEN1286 may suppress redundant signaling pathways and delay therapeutic resistance.

### 2.5. Targeted Therapies

#### 2.5.1. Receptor Tyrosine Kinase

Targeted therapies in NPC aim to disrupt key oncogenic pathways that regulate tumor growth, angiogenesis, immune evasion, and therapy resistance (Figure 1 and Table 1). Receptor tyrosine kinases (RTKs), including EGFR, vascular endothelial growth factor receptor (VEGFR), MET, and AXL, are key mediators of oncogenic signaling in NPC. For example, EGFR is frequently upregulated in NPC and correlates with disease progression, while VEGFR, MET, and AXL contribute to neovascularization and remodeling of the TME. Additionally, AXL has been associated with epithelial–mesenchymal transition and resistance to therapy [90].

Therapeutically, RTKs can be inhibited using monoclonal antibodies that block extracellular ligand binding or small-molecule tyrosine kinase inhibitors (TKIs) that interfere with intracellular ATP-binding sites. For example, the addition of nimotuzumab, an anti-EGFR antibody, to standard chemoradiation in locally advanced NPC demonstrated an acceptable safety profile and promising efficacy in multiple studies [91,92]. A phase III study, evaluating nimotuzumab in combination with toripalimab and chemotherapy as an induction regimen, is underway in locally advanced NPC. Similarly, pimurutamab, another anti-EGFR antibody, is currently under investigation for synergistic effects with chemotherapy or immune checkpoint blockade in R/M NPC.

VEGF, which plays a central role in tumor angiogenesis, has also been recognized as an important immune modulator in various solid tumors [93]. The blockade of the VEGFR/VEGF pathway using antagonistic antibodies or TKIs has been established as a standard cancer treatment. Simultaneous targeting of anti-PD1 and VEGF pathways has been suggested as a rational strategy. A combination of sintilimab and bevacizumab, an anti-VEGF antibody, demonstrated a potential signal of synergy with an ORR of 54.5% in platinum-refractory ICI-native R/M NPC [94]. Similarly, the combination of camrelizumab and apatinib, a VEGF receptor-2 TKI, showed promising efficacy with an ORR of 65% in post-platinum ICI-naïve and 43.3% in ICI-refractory R/M NPC patients [95].

#### 2.5.2. Other Targets

CDK4/6 inhibitors, such as dalpiciclib, not only suppress tumor proliferation through G1-phase arrest but also enhance tumor immunogenicity by upregulating MHC class I and interferon signaling, supporting their use in combination with immunotherapy [96]. The combination of dalpiciclib and camrelizumab showed efficacy in patients with anti-PD-1 refractory R/M NPC with an ORR of 32.4% (N = 34) [97]. Similarly, PARP inhibitors (e.g., niraparib, olaparib) exploit DNA repair deficiencies in NPC, inducing synthetic lethality and enhancing neoantigen exposure for immune recognition [98]. Clinical studies are ongoing to test this hypothesis in patients with advanced NPC, but the data are not yet available.

Epigenetic therapies targeting EBV latency offer novel therapeutic opportunities. Nanatinostat reactivates EBV lytic genes, enabling valganciclovir to be phosphorylated into a cytotoxic form that selectively kills infected tumor cells [99,100]. Inhibitor of apoptosis proteins, such as XIAP and cIAP1/2, also represent promising targets for restoring cell death and enhancing sensitivity to chemoimmunotherapy [101]. Among viral targets, EBNA1 plays a central role in maintaining EBV persistence by sustaining episomal replication and evading immune surveillance. Emerging inhibitors, such as VK-2019, impair EBNA1’s DNA-binding function, destabilizing episomes and reinstating antigen presentation, thereby priming tumors for immunotherapeutic attack [102,103]. However, it has a limited single-agent activity with one objective response seen out of 22 patients with R/M NPC

## 3. Summary and Outlook

Despite recent advancements, treatment options for advanced NPC remain limited. A primary challenge is the lack of clearly defined driver mutations that can be targeted with precision medicine [104]. Unlike other malignancies, where specific genetic alterations have led to the development of targeted therapies, NPC exhibits a more complex and heterogeneous genomic profile, complicating the identification of universal therapeutic targets [105,106]. Additionally, the relatively low incidence of NPC in Western countries has resulted in fewer clinical trials and limited pharmaceutical investment, contributing to this therapeutic stagnation [107].

However, the treatment landscape is evolving as drug development enters a new era, characterized by the introduction of diverse therapeutic modalities in oncology. ICIs targeting the PD-1/PD-L1 axis have shown promising efficacy in recurrent or metastatic disease [108]. Moreover, novel therapies, including bispecific antibodies, ADCs, therapeutic vaccines, and adoptive T-cell therapies, are actively being explored. These therapies aim to enhance treatment precision, overcome immune resistance, and exploit tumor-specific vulnerabilities. Robust research efforts, particularly in China and Southeast Asia, where disease burden is high, have accelerated therapeutic innovation in this domain [109].

A critical consideration in future therapeutic development is the biological distinction between EBV-associated NPC, which predominates in endemic regions, and non-EBV NPC, more commonly observed in Western populations [110]. This distinction can influence treatment response, particularly in immunotherapy, highlighting the need for tailored strategies and prospectively stratifying by EBV status Although immunotherapy has shown substantial promise, not all patients respond, and predictive biomarkers, such as PD-L1 expression, tumor mutational burden, and circulating EBV DNA, remain under investigation and require clinical validation [111,112]. The integration of new therapies into existing treatment regimens must also carefully consider toxicity profiles and potential interactions.

Additionally, an emerging body of work frames NPC as a dynamic ecosystem shaped by ecological and evolutionary processes [19,20]. perspectives argue that scheduling and adaptivity, rather than drug identity alone, can be crucial levers against resistance [113]. This framework supports prospective studies that incorporate adaptive or schedule-aware strategies, such as response-guided dosing intervals or radiotherapy schedules and exploitation of the ecological vulnerabilities and integrate longitudinal biomarkers to track evolutionary dynamics in NPC.

Despite these challenges, advancements in our understanding of the molecular and immunological landscape of the disease, combined with the development of novel therapies and biomarker-guided treatment strategies, hold the potential to significantly improve outcomes for patients with advanced NPC.

## Figures and Tables

**Table 1:** Novel agents in development for nasopharyngeal carcinoma.

**Immune Cell Activating Therapy**									
**Drug Name**	**Mechanism**		**Phase**	**NCT ID**	**Regimen**	**N**	**Population**	**Key Outcome**	**G ≥ 3 TRAE (%)**
Sintilimab	Anti-PD-1 mAb		III	NCT03700476	CCRT ± Sintilimab	425	LA-NPC	EFS HR 0.59; 36-mo EFS 86% vs. 76%	74
Envafolimab	Anti-PD-L1 mAb		II	NCT05397769	CCRT + Envafolimab	36	LA-NPC	ORR 94.4%	0
Serplulimab	Anti-PD-1 mAb		II	NCT05513573	Gem/Cis + Serplulimab	25	R/M NPC	ORR 72%; 12-mo PFS 52.8%	32
INCB099280	PD-L1 SMI		I	NCT04242199	Monotherapy	179	Adv. solid tumors	ORR 16.0%; mDOR 16.8 mo	13.4
Tagitanlimab	Anti-PD-L1 mAb		III	NCT05294172	Gem/Cis ± Tagitanlimab	358	R/M NPC	PFS HR 0.47; ORR 81.7%; mDOR 11.7 mo	3.9
IBI-310	Anti-CTLA-4 mAb		Ib/II	NCT04945421	IBI-310 + Sintilimab	30	R/M NPC	NR	NR
LBL-007	Anti-LAG-3 mAb		Ib/II	NCT05102006	LBL-007 + Toripalimab	30	R/M NPC	ORR 33.3%; mPFS 10.8 mo; mDOR 15 mo	11.3
Relatlimab	Anti-LAG-3 mAb		II	NCT06029270	Nivolumab ± Relatlimab	156	R/M NPC	NR	NR
TQB2618	Anti-TIM-3 mAb		II	NCT05563480	Penpulimab ± TQB2618	17	R/M NPC	ORR 0%; mPFS 1.6 mo; 6-mo PFS 18.2%	0
Tifcemalimab	Anti-BTLA mAb		I/II	NCT04929080	JS004 ± Toripalimab	149	R/M HNSCC and NPC	NR	NR
Bintrafusp alfa	PD-L1 × TGF-β fusion		II	NCT04396886	Monotherapy	38	R/M NPC	ORR 23.7%; mOS 17.0 mo; mPFS 2.3 mo;	42.4
Retlirafusp alfa	PD-L1 × TGF-β fusion		Ib	NCT04282070	Arm 1 (post-chemo) Arm 2 (post-PD-1)	54	R/M NPC	Arm 1: ORR 33.3%; mPFS 5.3 mo Arm 2: ORR 4.2%; mPFS 1.4 mo	18.5
TQB2858	PD-L1 × TGF-β fusion		Ib/II	NCT05198531	TQB2858 + Anlotinib	90	R/M NPC	NR	NR
Cadonilimab	PD-1 × CTLA-4 BsAb		II	NCT05790200	Cadonilimab + Chemo	25	PD-1-R R/M NPC	ORR 68%; mPFS 10.6 mo; mDOR 9.1 mo; 1-yr OS rate 75.6%	48
SI-B003	PD-1 × CTLA-4 BsAb		I	NCT04606472	Monotherapy	60	Adv. solid tumors	ORR 16.1%; mPFS 3.7 mo	3
QL1706	PD-1 × CTLA-4 BsAb		I	NCT04296994, NCT05171790	Monotherapy	110 (NPC)	Adv. solid tumors	ORR 24.5%; mDOR 11.7 mo (NPC cohort)	16
Vudalimab	PD-1 × CTLA-4 BsAb		I	NCT03517488	Monotherapy	77	Adv. solid tumors	ORR 13.0%	16.4
BGB-A445	Anti-OX40 agonist mAb		I	NCT04215978	Mono ± Tislelizumab	59/32	Adv. solid tumors	ORR 4%/23%	41/53
BAT6026	Anti-OX40 agonist mAb		I	NCT05105971	Monotherapy	30	Adv. solid tumors	ORR 0%; mPFS 1.5 mo	33.3
ADG106	Anti-4-1BB mAb		Ib/II	NCT04775680	ADG106 + Toripalimab	25	Adv. solid tumors	ORR 4.1%	16
ILB2109	A2AR SMI		Ib/IIa	NCT05955105	ILB-2109 + Toripalimab	200	Adv. solid tumors	NR	NR
DKY709	IKZF2 degrader		Ib	NCT03891953	DKY709 ± Spartalizumab	98	Adv. solid tumors	NR	NR
PLX-4545	IKZF2 degrader		I	ACTRN12623001265662	Monotherapy	NR	Adv. solid tumors	NR	NR
**Vaccines**									
**Drug Name**	**Mechanism**		**Phase**	**NCT ID**	**Regimen**	**N**	**Population**	**Key Outcome**	**G ≥ 3 TRAE (%)**
CD137L-DC-EBV-VAX	DC vaccine		I	NCT03282617	Monotherapy	12	R/M NPC	ORR 8.3%; mPFS 3.8 mo; mOS 20.8 mo	0
KSD-101	DC vaccine		I	NCT06370026, NCT06097793	Monotherapy	12	EBV^+^ NPC	NR	NR
DC-CIK	DC + CIK adoptive-cell vaccine		II	NCT01821495	CCRT ± DC-CIK	100	LA-NPC	NR	NR
Auto-DC ± Allo-DS	Autologous DC ± allogeneic dendritic-secretome		I/II	NCT05261750	RT/CCRT + Auto-DC ± Allo-DC	15	R/M NPC	NR	NR
MVA-EBNA1/LMP2	Viral-vector (MVA) vaccine		Ib	NCT01800071	Monotherapy	18	EBV^+^ NPC	83% responded to vaccine-coded antigens	0
VAC003	Viral-vector (MVA) vaccine		II	NCT01094405	Monotherapy	25	EBV^+^ R/M NPC	NR	NR
WGc-043	EBV-antigen mRNA vaccine		I	NCT05714748	Monotherapy	12	EBV^+^ R/M NPC	ORR 16.7%	0
**Adoptive Cell Therapy**									
**Therapy Type**	**Target Antigen(S)**		**Phase**	**NCT ID**		**N**	**Population**	**Key Outcome**	**G ≥ 3 TRAE (%)**
CTL (autologous)	LMP2, EBNA1		III	NCT02578641		330	R/M NPC	No benefit (chemo ± EBV CTL)	0.6
	LMP2, EBNA1		II	NCT00834093, NCT00431210		21	R/M NPC	ORR 4.8%; mPFS 2.2 mo; mOS 16.7 mo	0
	LMP1/2, BARF1, EBNA1		I	NCT02065362		14	EBV^+^ NPC	NR	NR
	Multi EBV Ag		I	NCT00608257		8	EBV^+^ R/M NPC	ORR 12.5%; EBV CTL + CD45	0
CTL (allogeneic)	EBV Ag (Tabelecleucel)		Ib/II	NCT03769467		12	EBV^+^ R/M NPC	SD 50%	0
CAR-T (autologous)	NR (U87)		I	NCT06614686		20	R/M HNSCC	NR	NR
	EBV gp350 (BRG01)		I	NCT05864924		11	EBV^+^ R/M NPC	Tumor shrinkage in 75%; PFS > 6 mo 100%	0
	NR		I	NCT05654077		24	R/M NPC	NR	NR
	LMP1		I/II	NCT02980315		20	EBV-associated tumors	NR	NR
	EpCAM		I	NCT02915445		12	EpCAM^+^ solid tumors	ORR 16.7%	8.3
	Dual EBV Ag (BGT007)		I	NCT05616468		23	R/M NPC	NR	NR
CAR-T (allogenic)	CD70 (CHT101)		I	NCT06383507		18	Adv. solid tumors	NR	NR
	MUC1-C (P-MUC1C-ALLO1)		I	NCT05239143		6	Adv. solid tumors	ORR 16.7%	0
CAR-T (allogenic, γδ)	NKG2DL (CTM-N2D)		I	NCT04107142		10	Adv. solid tumors	NR	NR
TCR-T	EBV Ag		I/II	NCT04509726		20	EBV^+^ R/M NPC	NR	NR
	LMP1/2, EBNA1 (YT-E001)		II	NCT03648697		20	EBV^+^ R/M NPC	NR	NR
	LMP2		I	NCT03925896		27	EBV^+^ R/M NPC	NR	NR
Mixed CAR-T/TCR-T	EBV Ag		I	NCT05587543		24	EBV^+^ R/M NPC	NR	NR
TIL (autologous)	NR		I	NCT01462903		20	LA-NPC	ORR 95%	5
Allo-CTL	EBV Ag + Pembrolizumab + Tabelecleucel		I/II	NCT03769467		12	EBV^+^ R/M NPC	ORR 0%	0
**Antibody Drug Conjugate**									
**Drug**	**Target**	**Payload**	**Phase**	**NCT ID**	**Regimen**	**N**	**Population**	**Key Outcome**	**G ≥ 3 TRAE (%)**
Becotatug vedotin	EGFR	HY-15162	IIa	NCT05126719	Monotherapy	61	R/M NPC (post Pt/PD-1)	ORR: 39.3% (DL1), 55.2% (DL2);	11.5
			I/II	NCT05688605	MRG003 + HX008	30	Adv. solid tumors	ORR 66.7%, 6-mo PFS rate 76.2% (NPC sub-cohort, *n* = 9)	23.3
GEN1160	CD70	DX-8951	I/II	NCT05721222	Monotherapy	134	R/M NPC, RCC, NHL	NR	NR
YL201	B7-H3	YL0010014	I/II	NCT05434234 /NCT06057922	Monotherapy	312	Adv. solid tumors	ORR 48.6%; mPFS: 7.8 mo (NPC sub-cohort, *n* = 70))	54.5
TAK-500	CCR2	TAK-676	I/II	NCT05070247	TAK-500 ± Pembrolizumab	61	Adv. solid tumors	NR	NR
BL-B01D1	EGFR x HER3	Ed-04	I	NCT05194982	Monotherapy	195	Adv. solid tumors	ORR 38%, mPFS 6.8 mo (NPC sub-cohort, *n* = 42)	71
GEN1286	EGFR x MET	DX-8951	I/II	NCT06685068	Monotherapy	260	Adv. solid tumors	NR	NR
**Target Therapy**									
**Drug**	**Molecular Target**	**Class**	**Phase**	**NCT ID**	**Regimen**	**N**	**Population**	**Key Outcome**	**G ≥ 3 TRAE (%)**
Nimotuzumab	EGFR	mAb	III	NCT06561763	Toripalimab + Nimotuzumab	416	LA-NPC	NR	NR
Pimurutamab	EGFR	mAb	II	NCT05513573	Pimurutamab + HLX10 + Chemo	75	R/M NPC	ORR 72%; 12-mo PFS 63.0%	28.0
Anlotinib	VEGFR/FGFR/PDGFR/KIT/RET	TKI	II	NCT03906058	Monotherapy	39	R/M NPC	ORR 20.5%; mPFS 5.7 mo	23.7
Cabozantinib	VEGFR/MET/RET/KIT/Tie2/AXL/FLT3	TKI	II	NCT05904080	Nivolumab/Ipilimumab ± Cabozantinib	50	R/M NPC	NR	NR
Apatinib	VEGFR2	TKI	II	NCT04586088	Apatinib + Camrelizumab	58	R/M NPC	ORR 65.5%; mPFS 10.4 mo	58.6
Surufatinib	VEGFR/FGFR1/CSF-1R	TKI	II	NCT04955886	Surufatinib + Toripalimab	14	R/M NPC	NR	NR
Axitinib	VEGFR/PDGFRβ/KIT	TKI	II	NCT01249547	Monotherapy	40	R/M NPC	mOS 10.4 mo, 1-yr OS rate 45.4%	8
		TKI	II	NCT04562441	Axitinib + Avelumab (PD-L1)	13	ICI-naïve R/M NPC	ORR 7.7%; mPFS 5.4 mo, mOS 15.0 mo	NR
Dalpiciclib	CDK4/6	SMI	II	NCT05724355	Dalpiciclib + Camrelizumab	34	R/M NPC (PD-1 resistant)	ORR 32.4%; mDOR 10.4 mo, mPFS 6.7 mo	76.5
Niraparib	PARP	SMI	II	NCT05162872	Niraparib + Sintilimab	99	R/M NPC	NR	NR
Fuzuloparib	PARP	SMI	II	NCT04978012	Fuzuloparib + Camrelizumab	48	R/M NPC	NR	NR
Olaparib	PARP	SMI	III	NCT04825990	Olaparib + Pembrolizumab	34	R/M NPC (Pt resistant)	ORR 13%; mPFS 4 mo	22
Nanatinostat	HDAC	HA	I	NCT05166577	Nanatinostat + Valganciclovir	15	EBV^+^ R/M NPC	ORR 6.7%	0
Tolinapant	cIAP1/2, XIAP	NPM	I	NCT05245682	Tolinapant + RT	10	LA-HNSCC	NR	0
Taladegib	SMO (Hh)	SMI	II	NCT05199584	Monotherapy	44	PTCH1-mutated solid tumors	NR	NR
VK 2019	EBNA1	SMI	II	NCT04925544	Monotherapy	22	EBV^+^ R/M NPC	ORR 4.5%	NR

A2AR, adenosine A2A receptor; BsAb, bispecific antibody; BTLA, B- and T-lymphocyte attenuator; CAR-T, Chimeric antigen receptor T cells; CCRT, concurrent chemoradiotherapy; CDK, Cyclin-dependent kinase; CIK, cytokine-induced killer cells; cIAP/XIAP, cellular/X-linked inhibitor of apoptosis protein; CSF-1R, Colony-stimulating factor 1 receptor; CTL, cytotoxic T lymphocyte; CTLA-4, cytotoxic T-lymphocyte-associated antigen 4; DC, dendritic cell; EBNA1, Epstein–Barr virus nuclear antigen 1; EBV, Epstein–Barr virus; EGFR, Epidermal growth factor receptor; EFS, event-free survival; FGFR, Fibroblast growth factor receptor; Gem/Cis, gemcitabine + cisplatin; HA, hydroxamic acid; HDAC, Histone deacetylase; HNSCC, head and neck squamous cell carcinoma; ICI, immune-checkpoint inhibitor; IKZF2, Ikaros family zinc finger 2; LA-NPC, locally advanced nasopharyngeal carcinoma; LAG-3, lymphocyte-activation gene-3; mAb, monoclonal antibody; mDOR, median duration of response; MET, Mesenchymal–epithelial transition factor; mo, months; mOS, median overall survival; mPFS, median progression-free survival; MVA, modified vaccinia Ankara; NPC, nasopharyngeal carcinoma; NPM, non-peptidomimetic; NR, not reported; PARP, Poly(ADP-ribose) polymerase; PD-1, programmed cell death protein-1; PD-L1, programmed death-ligand 1; PDGFR, Platelet-derived growth factor receptor; Pt, platinum; PTCH1, Protein patched homolog 1; R, resistant; R/M NPC, recurrent/metastatic NPC; RT, radiotherapy; SD, stable disease; SMI, small-molecule inhibitor; SMO, Smoothened receptor (Hedgehog pathway); TCR, T-cell receptor; TCR-T, T-cell receptor-engineered T cells; TGF-β, transforming growth factor-β; Th1/Tc1, type-1 helper/cytotoxic T cell; TIL, Tumor-infiltrating lymphocytes; TIM-3, T-cell immunoglobulin and mucin-domain containing-3; TKI, tyrosine–kinase inhibitor; TNFα, Tumor necrosis factor-alpha; TRAE, treatment-related adverse event; VEGFR, Vascular endothelial growth factor receptor.
